# Older Adults’ Subjective Well-being Following an Exergame Intervention “Bandit the Dolphin”: Focus Group Findings

**DOI:** 10.1093/geroni/igaa057.549

**Published:** 2020-12-16

**Authors:** Brittany Drazich, Breanna Crane, Kyle Moored, Karl Shieh, Janiece Taylor, Michelle Carlson

**Affiliations:** 1 Johns Hopkins School of Nursing, Baltimore, Maryland, United States; 2 Johns Hopkins Bloomberg School of Public Health, Baltimore, Maryland, United States; 3 Johns Hopkins School of Public Health, Baltimore, Maryland, United States; 4 Johns Hopkins University, Johns Hopkins University, Maryland, United States

## Abstract

Due to generational mental illness stigma and under diagnosis of mental illness, older adults do not always receive the mental health help that they need. One unique technology that has the potential to improve mood in older adults is exergames, or exercise video games. The objective of this sub-study (main study: Stimulation With Intricate Movements “SWIM” Study) was to explore older adults’ mood following an exergame intervention called “Bandit the Dolphin,” created by the Johns Hopkins KATA Studio. Researchers conducted three focus groups with 14 community-dwelling older adult participants who took part in the SWIM Study exergame intervention. The semi-structured focus groups were transcribed, coded, and analyzed using deductive and inductive techniques described by Ray Maietta’s “sort and sift, think and shift” method. Three themes related to playing “Bandit the Dolphin” and mood emerged. First, participants described their perceived association between activity and mood. Participants felt that both active and passive activities, “Bandit the Dolphin” and otherwise, improved their mood through the “fun” factor, and through feelings of achievement. Second, the participants described that the competition and frustration of playing “Bandit the Dolphin” increased eventual feelings of achievement. Third, participants described how feelings of immersion, or being absorbed in the game, helped them forget their other life concerns. These findings provide a better understanding of older adults’ perceived relationship between an exergame intervention, “Bandit the Dolphin,” and short-term improved mood. Future health and engineering researchers should explore exergames as a potential tool to improve the mental health of older adults.

